# Changes in attitudes towards smoking during smoking cessation courses for Turkish- and Albanian-speaking migrants in Switzerland and its association with smoking behavior: A latent change score approach

**DOI:** 10.3389/fpsyg.2022.1032091

**Published:** 2022-12-22

**Authors:** Raquel Paz Castro, Mirka Henninger, Michael P. Schaub, Corina Salis Gross

**Affiliations:** ^1^Swiss Research Institute for Public Health and Addiction at the University of Zurich, Zurich, Switzerland; ^2^Marie Meierhofer Institut für das Kind, Zurich, Switzerland; ^3^Psychological Methods, Evaluation and Statistics, Department of Psychology, University of Zurich, Zurich, Switzerland

**Keywords:** migrants, immigrants, smoking cessation, behavior change, attitudes, latent change score

## Abstract

**Introduction:**

Migrant populations usually report higher smoking rates than locals. At the same time, people with a migration background have little or no access to regular smoking cessation treatment. In the last two decades, regular smoking cessation courses were adapted to reach out to Turkish- and Albanian-speaking migrants living in Switzerland. The main aims of the current study were (1) to analyze the effects of an adapted smoking cessation course for Turkish- and Albanian-speaking migrants in Switzerland on attitudes toward smoking and smoking behavior; and (2) to elucidate whether changes in attitudes toward smoking were associated to changes in smoking behavior in the short- and in the long-term.

**Methods:**

A total of 59 smoking cessation courses (Turkish: 37; Albanian: 22) with 436 participants (T: 268; A: 168) held between 2014 and 2019 were evaluated. Attitudes toward smoking and cigarettes smoked per day were assessed at baseline and 3-months follow-up. One-year follow-up calls included assessment of cigarettes smoked per day. Data were analyzed by means of structural equation modeling with latent change scores.

**Results:**

Participation in an adapted smoking cessation course led to a decrease of positive attitudes toward smoking (T: *β* = −0.65, *p* < 0.001; A: *β* = −0.68, *p* < 0.001) and a decrease of cigarettes smoked per day in the short-term (T: *β* = −0.58, *p* < 0.001; A: *β* = −0.43, *p* < 0.001) with only Turkish-speaking migrants further reducing their smoking in the long-term (T: *β* = −0.59, p < 0.001; A: *β* = −0.14, *p* = 0.57). Greater decreases in positive attitudes were associated with greater reductions of smoking in the short-term (T: *r* = 0.39, *p* < 0.001; A: *r* = 0.32, *p* = 0.03), but not in the long-term (T: *r* = −0.01, *p* = 0.88; A: *r* = −0.001, *p* = 0.99).

**Conclusion:**

The adapted smoking cessation courses fostered changes in positive attitudes toward smoking that were associated with intended behavior change in the short-term. The importance of socio-cognitive characteristics related to behavior change maintenance to further increase treatment effectiveness in the long-term is discussed.

## Introduction

Smoking is a modifiable leading risk of disease (Benziger et al., 2016). Current reviews on smoking cessation interventions have found that mass media campaigns, mobile phone-based interventions, nicotine replacement therapy (NRT), and behavioral therapies in groups or single sessions to be effective for smoking cessation (Whittaker et al., 2016; Bala et al., 2017; Lancaster and Stead, 2017; Stead et al., 2017; Hartmann-Boyce et al., 2018; Rigotti et al., 2022). However, few of the included studies focused on migrant populations and still little is known about mechanisms responsible for changing smoking habits (Baker and Mccarthy, 2021). From existing reviews focusing on minorities it is evident that adapted smoking cessation interventions are more accepted (Liu et al., 2013) and can be more effective than non-adapted ones when aspects, such as family level, are considered (Nierkens et al., 2013).

Migrant populations usually report higher smoking rates, as evident in certain European countries (Lindstrom and Sundquist, 2002; Reeske et al., 2009; Brathwaite et al., 2017; Salama et al., 2018). From the latest health monitoring on migrants living in Switzerland (Guggisberg et al., 2011) it is known that daily smoking prevalence rates are higher for people that migrated from Turkey (men: 55.2%, women: 29.5%), and Kosovo (men: 33.7%, women: 24.2%) than for the Swiss general population (men: 21.7%, women: 19.5%), however, migrants living in Switzerland have hardly access to regular smoking cessation services (Spiess and Schnyder-Walser, 2018). The higher smoking rates can mainly be explained by the higher probability of migrants suffering from one of the so-called “social determinants of health” (e.g., poorer socio-economic status, being unmarried or cohabiting, unemployment, lower education level; Guggisberg et al., 2011; Reiss, 2013; Salama et al., 2018; Baker and Mccarthy, 2021).

As interventions can hardly change those determinants, the focus relies on cognitive (i.e., stage of readiness, attitudes, self-efficacy, norms, risk perception) or psychosocial factors (i.e., stress, negative affectivity), which are also known for their impact on smoking outcomes (DiClemente et al., 1985; Etter et al., 2000; Ferrer and Klein, 2015; Cramm and Nieboer, 2018). Depending on the current stage of behavior change of the smoker (pre-intentional, intentional, action) some variables are more relevant in order to accelerate the process than others are.

The importance of attitudes (advantages and disadvantages of a health behavior) is widely discussed in known theories of behavior change, such as the theory of planned behavior (Ajzen, 1991), the transtheoretical model (Prochaska and Velicer, 1997), health action process approach (Schwarzer, 1992), the I-change model (De Vries, 2017) and have also been evaluated for migrant populations (Marin et al., 1990; Kramer Lafferty et al., 1999; Nierkens et al., 2005). Typically, these theories hypothesize that the advantages of smoking must decrease while the disadvantages should increase. However, studies mostly found perceived advantages, not disadvantages, to be predictive for smoking outcomes (Prochaska and Velicer, 1997; Etter et al., 2000; Nierkens et al., 2005; Khazaal et al., 2013; Hoeppner et al., 2019). The most common explanation for these findings is that daily smokers already have a relatively good awareness related to the adverse effects of smoking and it is probably difficult to increase this aspect (Etter et al., 2000; Khazaal et al., 2013).

Only one of the included studies was conducted on Middle Eastern migrants groups, such as Turks and Moroccans (Nierkens et al., 2005). Further, most of them based their conclusions on cross-sectional data (Marin et al., 1990; Prochaska and Velicer, 1997; Kramer Lafferty et al., 1999; Etter et al., 2000; Nierkens et al., 2005). To the best of our knowledge, no study has tested if a smoking cessation intervention was able to change attitudes toward smoking in migrants and thereby foster behavior change. The main aims of the current study are (1) to analyze the effects of an adapted smoking cessation course for Turkish- and Albanian-speaking migrants in Switzerland on attitudes toward smoking and smoking behavior; and (2) to elucidate whether changes in attitudes toward smoking are associated to changes in smoking behavior in the short- (T2) and in the long-term (T3).

## Materials and methods

### Smoking cessation courses for Turkish- and Albanian-speaking migrants

In as early as 2006, group cessation courses for Turkish-speaking migrants living in Switzerland were developed and implemented to foster health equity (Schnoz et al., 2011). In 2010, the adapted smoking cessation courses were integrated into the National Tobacco Prevention Program, and in 2016, the approach was multiplied to Albanian-speaking migrants living in Switzerland. The effectiveness of the group cessation courses for Turkish-speaking migrants living in Switzerland has been evaluated twice, first for the initial courses (Schnoz et al., 2011) and second over the implementation period of 2006 until 2018 (Paz Castro et al., 2021). Of the 478 Turkish-speaking migrants who participated in smoking-cessation courses from 2006 until 2018, 45.4% declared themselves non-smokers at 1-year follow-up. This smoking cessation rate was higher than the one achieved in the preliminary evaluation of the program on 61 participants (37.7%) and comparable to the last evaluation of the smoking cessation courses aimed at the local (Swiss) population (43.9%; Schmid et al., 2012). Characteristics associated with long-term abstinence were length of the course (eight vs. six sessions), adherence to the course, use of pharmacotherapy or NRT products, and baseline level of dependence.

For the present study, data from the courses with Turkish-speaking migrants from 2014 onwards will be used since measures relevant to the study’s aims, such as attitudes toward smoking, were not assessed in earlier courses. Compared to previous studies, data from the Turkish as well as from the courses with Albanian migrants, which started in 2016, will be examined.

The development of the smoking cessation program for Turkish-speaking migrants in Switzerland has previously been described in detail (Schnoz et al., 2011; Paz Castro et al., 2021). In short, the smoking cessation courses for the Turkish- and Albanian-speaking migrants are grounded in behavioral therapy and were adapted from the weekly group-counseling sessions applied by the Cancer League Zurich. The main aim of the smoking cessation courses was identical for both groups, namely to hold a collective quit day between the second and the third group session. Before quit day, information about the harmful effects of smoking on the human body as well as explanations about craving symptoms during smoking cessation and alternatives to smoking were discussed. On quit day, the positive connotation of cessation (not a loss, but a gain in health and quality of life) was enhanced and basic relapse prevention (Nicotine Replacement Therapy, National Quit Smoking Helpline) was discussed. After quit day, relapse prevention was intensified and strategies for weight control were discussed. At each of the six weekly group sessions, the level of carbon monoxide (CO) in the breath of every participant was analyzed with a piCO smokerlyzer (Bedfont Scientific Ltd.).

The adaptation of the course material was based on social, cultural, economic factors as well as language and health literacy (Nutbeam, 2000; Institute of Medicine (US) Committee on Health Literacy, 2004). Key members of the Turkish, Kurdish and Albanian communities living in Switzerland double-checked the adaptations and insured that symbolic references to typical Swiss situations were replaced with typical lifeworld-situations of the Turkish or Albanian immigrants (i.e., replacing typical smoking situations for Swiss people with typical smoking situations for Turkish-or Albanian-speaking people living in Switzerland, which were the only minor differences that emerged for both groups). Besides that, the adaptation included the possibility to offer women’s only and men’s only courses, and in so doing, respect gender roles and gender-lifeworld. Lastly, the courses were offered free of charge in contrast to those applied by the Cancer League Zurich. This adaptation was necessary to increase the reach of migrants with low socioeconomic status.

### Participants and recruitment

A multi-modal strategy of subject recruitment was pursued. Initially, the smoking cessation coaches held an informative talk at clubhouses or organizations and religious institutions of the Diaspora in Switzerland to inform all interested persons about the hazards of smoking. With these talks, not only smokers, but also non-smokers possibly affected by the smoking behavior of a relative were reached. A total of 123 talks (Turkish: 75; Albanian: 48) were held between 2014/2016 and 2019 with a reach of approximately 4′459 (Turkish: 3′494; Albanian: 965) persons. After the talks, key members of the community or of informal groups helped the smoking cessation coaches to recruit smokers interested in a cessation course.

Subjects were also recruited by the smoking cessation coaches at a variety of events surrounding the Turkish- and Albanian-speaking community in Switzerland (e.g., mosques) with adapted posters and flyers. Further, personal networks of the coaches were used to reach smokers and form cessation courses. Additionally, advertisements were distributed *via* different media (internet and print), local radio stations, national Turkish and Albanian television and, most recently, Facebook. Once a group consisting of 6 to 8 people was formed, the smoking cessation coach rented a location that was similarly accessible for all participants. Together with the group, the course dates and hours were defined.

The inclusion criterion for participation on a cessation course was smoking cigarettes at any level the month previous to the course start. Sufficient or insufficient mastery of the language of the host country was not an inclusion/exclusion criterion since the courses were held in their mother tongue. A total of 59 courses (Turkish: 37; Albanian: 22) with 436 participants (Turkish: 268; Albanian: 168) were formed from 2014/2016 to 2019. All subjects smoked cigarettes at least monthly and were thus eligible for the study. Eligible persons were informed about the study purpose, that they could cancel their participation at any time without negative consequences, and that all of their data would be treated confidentially. All 436 course participants accepted to take part in the study. For further analysis, a key coding strategy was followed. Key codes were kept securely by the principal investigator of the study. The collection of the data was revised and approved by the Tobacco Control Fund at Federal Office of Public Health Switzerland. The study does not include a clinical trial but a self-evaluation of a program which is part of the Swiss Public Health, which is why the Swiss Human Research Act (HRA) of 2014, regulating research on human participants, did not apply. The HRA regulates which projects have to undergo a review process by an ethics committee in Switzerland. The evaluation of the program was executed in compliance with the Helsinki Declaration.

### Measures and outcomes

To analyze the effects of an adapted smoking cessation course, participants took part in several assessments, including a pre-questionnaire at beginning of session 1 (T1), a post-questionnaire *via* telephone 3 months after quit day (T2, approximately 8 weeks after the last group session), and a follow-up telephone call 12 months (T3) after the quit day.

Trained interviewers who were unknown to the participants and spoke their mother tongue (Turkish or Albanian) conducted both the 3-month assessment and 1-year follow-up telephone call.

The pre/post questionnaires at T1 and T2 included the socio-cognitive variables ‘attitudes toward smoking’ and tobacco-related variables, such as smoking status, and number of cigarettes smoked per day (CPD).

Measures included only in T1 were socio-demographic and socio-cognitive variables. *Socio-demographic variables* were gender, age, marital status, parental status, additional Swiss nationality, educational attainment, employment status, age at first contact with smoking, and age of onset of regular smoking. *Socio-cognitive variables* were self-efficacy, perceived stress and perceived environmental.

*Attitudes toward smoking* were measured by eight beliefs about the perceived positive and negative consequences of smoking on a four-point Likert-scale with the response format from 1 “strongly disagree” to 4 “strongly agree”. Using confirmatory factor analysis (for more details see below), two subscales were extracted: *positive attitudes toward smoking* with the three items “Smoking helps against boredom”, “Smoking calms and relaxes”, and “Smoking tastes good” (α = 0.68) and *negative attitudes toward smoking* with the three items “Smoking makes the skin age faster”, “Smoking leaves an unpleasant smell”, and “Smoking damages the health of other people” (α = 0.68). The two items “Smoking underlines a modern attitude” and “What is your opinion on the general ban on smoking in public places, restaurants, cafés and bars?” were unsubstantial and were excluded of further analysis.

*Smoking behavior* was measured by number of cigarettes smoked per day (CPD) and was assessed at pre- (T1), post- (T2), and follow-up (T3) assessment.

### Data analysis

#### Invariance tests of migrant and gender groups

In a first step, confirmatory factor analyses (CFA) were conducted on the subscales positive and negative attitudes toward smoking to confirm the factorial structure on data from T1 and T2. In a second step, a series of invariance restrictions were tested for the subscales positive and negative attitudes toward smoking across migrant groups (Turkish- vs. Albanian-speaking migrants) and gender within migrant groups, before proceeding to test longitudinal invariance. We conducted the invariance test because first, it needs to be ensured that the observed measures are understood in the same way by the different migrant groups and gender subgroups and second, it needs to be ensured that the observed measures were understood the same way at every measurement occasion (Bereiter, 1963). The invariance tests included configural, metric and scalar restrictions (Meredith, 1993). These restrictions were tested separately for T1 and T2 as well as longitudinally on the level of the migrant group (Turkish/Albanian). If invariance requirements were not met for migrant groups, we tested gender invariance within the migrant groups (Hu and Bentler, 1995).

#### Latent change score models

**Figure 1:**
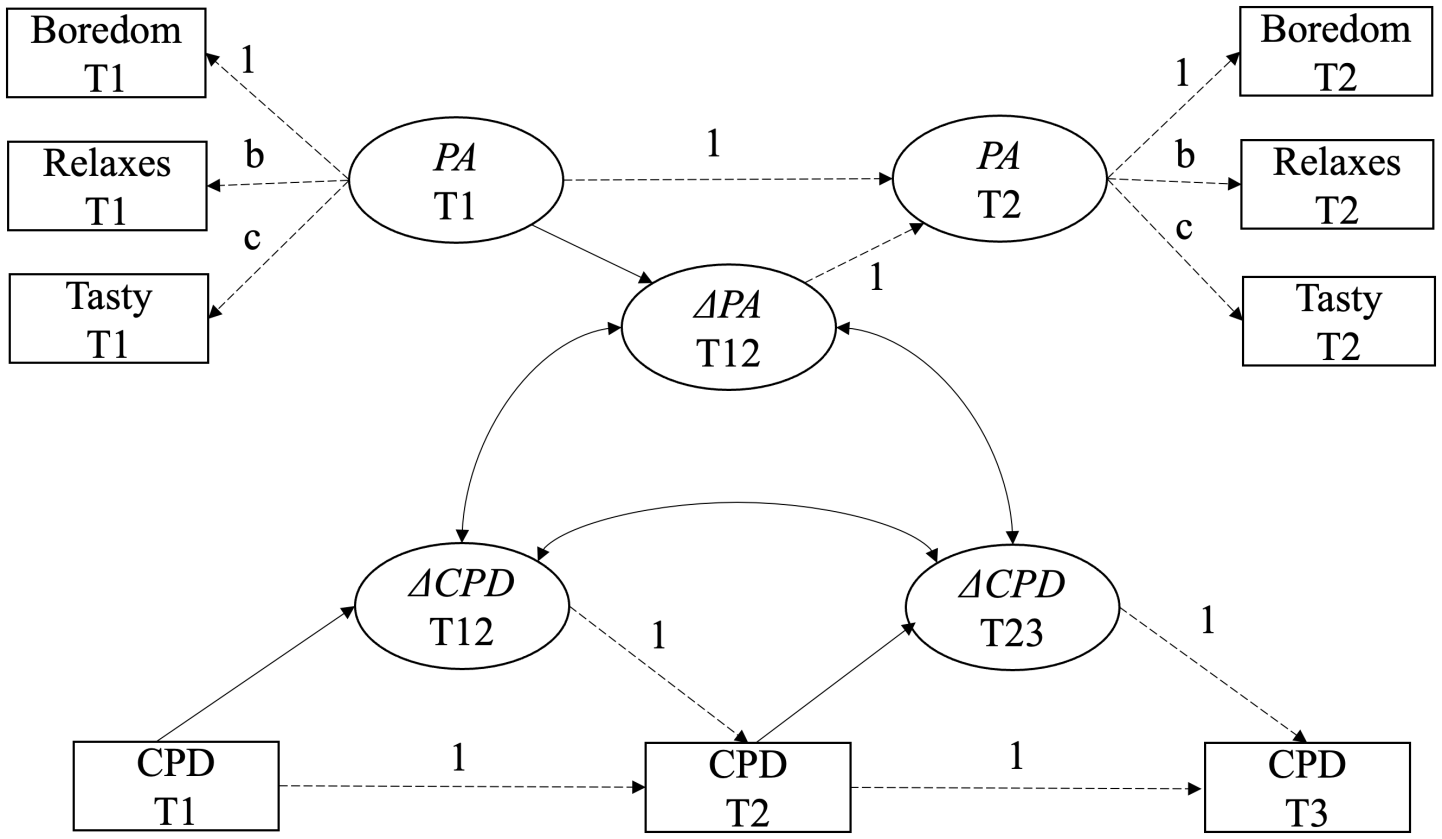
Specified latent change score model for changes in positive attitudes (PA) and its associations with changes in cigarettes smoked per day (CPD) between pre- (T1), post- (T2), and follow-up (T3) assessment. Δ symbolizes latent change scores; dashed arrows reflect fixed or constrained paths. Cross-time correlations between the same items across measurement occasions are left out to enhance visibility.

If measurement invariance was ensured for a specific migrant group or gender subgroup as well as longitudinally, we specified latent change score models to examine changes in attitudes toward smoking over time and its associations with changes in how many cigarettes were smoked per day. Latent change score models allowed us to test whether, besides a linear effect, change also depends on the previous time point (Ghisletta and McArdle, 2012). In addition, with latent change score models, typical problems of observed difference scores, for example their low reliability (Plewis, 1985), can be circumvented. An example of the latent change score model specified for positive attitudes is depicted in Figure 1. Latent factors of positive attitudes (PA) are defined based on three indicators at the two time points (T1 and T2) with factor loadings for the indicators being constrained to be equal across time. To account for multiple assessment of smoking attitudes, we introduced cross-time correlations between the same item across occasions (Jöreskog, 1979). The change score (ΔPA T12) is defined by regressing PA T2 on PA T1 with a fixed regression weight of 1 so one can emulate which part of PA T2 is not the same as PA T1 (McArdle, 2009). In consequence, ΔPA T12 directly represents a latent difference between the two time points. Two change scores for cigarettes smoked per day (ΔCPD T12 and ΔCPD T23) were specified the same way. The variance of the change scores indicated interindividual differences in intraindividual change with respect to positive attitudes and smoking behavior. In order to examine whether changes in positive attitudes toward smoking influence smoking behavior, we examined latent correlations between change scores (ΔPA T12, ΔCPD T12, and ΔCPD T23) as indicated by the double-pointed arrows in the structural model.

All models were estimated using the lavaan package (Rosseel, 2012) in R (R Core Team, 2018) using the WLSMV estimator which is appropriate for ordered categorical outcomes. Furthermore, sensitivity analyses were performed to estimate effects of the Covid-19 pandemic on changes in smoking behavior. Model fit was evaluated by means of the following goodness-of-fit-indices: (1) Chi-square goodness of fit test, (2) Comparative Fit Index (CFI), (3) the root mean square error of approximation (RMSEA), and (4) standardized root mean square residuals (SRMR) using the following criteria: when CFI > 0.95, RMSEA < 0.06, and SRMR < 0.08 models were considered to have a good fit, when CFI between 0.90 and 0.95 and RMSEA < 0.08 models were considered to have an acceptable fit (Hu and Bentler, 1995). As nested models were used for invariance tests, measurement invariance was assumed if a chi-square test indicated no significant differences between the restricted and less restricted model.

## Results

### Participants’ baseline characteristics

Baseline characteristics of the study sample are summarized in Table 1. The majority of participants were daily smokers (86.2%) and reported a mean consumption of almost one cigarette pack per day. With respect to the so-called “social determinants of health,” almost one third of the sample was characterized by higher psychosocial vulnerability, like lower education level (26.4%), being unemployed (27.8%) or being divorced/separated, widowed or living apart (15.2%). The Turkish and Albanian sample differed significantly in most baseline characteristics, except for number of cigarettes smoked per day, age of smoking onset, and positive attitudes toward smoking at baseline (*cf.* Table 1).

**Table 1:** Baseline characteristics of the full study sample.

		Overall*N* = 436	Turkish*n* = 268	Albanian*n* = 168	*p**^a^*
Sex (%)	Male	197 (45.2)	132 (49.3)	65 (38.7)	0.02
	Female	220 (50.5)	123 (45.9)	97 (57.7)	
	Missing	19 (4.4)	13 (4.9)	6 (3.6)	
Age, M (SD)^b^		43.6 (11.8)	44.7 (11.5)	41.8 (12.2)	0.02
Marital status (%)	Single	69 (15.8)	49 (18.3)	20 (11.9)	0.01
	Married/stable partnership	276 (63.3)	156 (58.2)	120 (71.4)	
	Married and living apart	16 (3.7)	11 (4.1)	5 (3.0)	
	Divorced/separated	37 (8.5)	31 (11.8)	6 (3.6)	
	Widowed	13 (3.0)	7 (2.6)	6 (3.6)	
	Missing	25 (5.7)	14 (5.2)	11 (6.5)	
Children living in same household (%)	No	161 (36.9)	116 (43.3)	45 (26.8)	0.00
	Yes	252 (57.8)	138 (51.5)	114 (67.9)	
	Missing	23 (5.3)	14 (5.2)	9 (5.4)	
Swiss nationality (%)	No	254 (58.3)	184 (68.7)	70 (41.7)	0.00
	Yes	128 (29.4)	73 (27.2)	55 (32.7)	
	Missing	54 (12.4)	11 (4.1)	43 (25.6)	
Highest education level (%)	No school attended	21 (4.8)	18 (6.7)	3 (1.8)	0.00
	Primary school (years 7–12)	94 (21.6)	60 (22.4)	34 (20.2)	
	Middle school (years 12–15)	94 (21.6)	45 (16.8)	49 (29.2)	
	Upper school (years 15–18)	132 (30.3)	92 (34.3)	40 (23.8)	
	University (years 18+)	68 (15.6)	37 (13.8)	31 (18.5)	
	Missing	27 (6.2)	16 (6.0)	11 (6.5)	
Working status (%)	Yes, full-time	120 (27.5)	52 (19.4)	68 (40.5)	0.00
	Yes, part-time	74 (17.0)	34 (12.7)	40 (23.8)	
	Housewife	36 (8.3)	29 (10.8)	7 (4.2)	
	In education	18 (4.1)	13 (4.9)	5 (3.0)	
	No	121 (27.8)	98 (36.6)	23 (13.7)	
	Missing/no comment	67 (15.4)	42 (15.6)	25 (14.8)	
Tobacco smoking status (%)	Daily smoker	376 (86.2)	240 (89.6)	136 (81.0)	0.00
	Occasional smoker	37 (9.0)	12 (4.5)	25 (14.9)	
	Missing	21 (4.8)	16 (5.9)	7 (4.2)	
Number of cigarettes smoked per day (CPD), *M* (*SD*)^c^		16.2 (9.3)	16.8 (8.9)	15.2 (9.9)	0.09
Age of first contact with smoking, *M* (*SD*)^d^		17.6 (5.4)	17.2 (4.9)	18.3 (6.0)	0.04
Age of onset of regular smoking, *M* (*SD*)^e^		20.7 (7.0)	20.5 (7.1)	20.9 (6.9)	0.65
Positive attitudes toward smoking, *M* (*SD*)^f^		2.5 (0.8)	2.5 (0.8)	2.6 (0.8)	0.53
Negative attitudes toward smoking, *M* (*SD*)^g^		3.7 (0.6)	3.8 (0.4)	3.5 (0.7)	0.00

a *p*-values for the comparison of the Turkish-speaking vs. Albanian-speaking group; χ^2^ tests for categorical variables, *t*-tests for metric variables.

b Missing information *n* = 42.

c Missing information *n* = 25.

d Missing information *n* = 35.

e Missing information *n* = 85.

f Missing information *n* = 78.

g Missing information *n* = 53.

### Attrition

At 3-month assessment (T2), 76.6% (334 out of 436) of the course participants were reached, whereas the reach was even higher at 1-year follow-up (T3; 85.6%, 373 out of 436).

Attrition analysis revealed that 3-month assessments were more likely to be completed by Turkish-speaking than Albanian-speaking migrants (*χ^2^* = 41.4, *df* = 1, *p* < 0.001), by male than female participants (*χ^2^* = 6.6, *df* = 1, *p* = 0.01), and by participants who reported lower ratings on positive attitudes (*t* = −2.0, *df* = 381, *p* = 0.05) and by those with higher ratings in negative attitudes toward smoking at baseline (*t* = 2.3, *df* = 381, *p* = 0.004).

Attrition analysis revealed that 1-year follow-ups were more likely to be completed by Turkish-speaking than Albanian-speaking migrants (*χ^2^* = 36.9, *df* = 1, *p* < 0.001), by male than female participants (*χ^2^* = 4.9, *df* = 1, *p* = 0.03), by participants who smoked daily (*χ^2^* = 4.6, *df* = 1, *p* = 0.03), and by participants who reported higher ratings in negative attitudes toward smoking at baseline (*t* = 2.9, *df* = 52.9, *p* = 0.004).

### Invariance of attitudes toward smoking across migrant groups, gender, and time

First, measurement invariance was tested separately for positive attitudes of smoking across migrant groups at T1 and T2. Changes in χ*^2^* from configural to metric invariance were significant. In other words, the factor structures differed for Turkish- and Albanian-speaking persons. Whereas Turkish-speaking persons saw the biggest advantage of smoking in its relaxing effect, Albanian-speaking persons saw the biggest advantage of smoking in their use against boredom.

Based on these results, further invariance testing was performed for both migrant groups separately. Measurement invariance for positive attitudes toward smoking was given for Turkish-speaking and Albanian-speaking women and men separately at T1, T2 and over time [Fit indices for longitudinally invariance with scalar restrictions: Turkish: χ*^2^* = 3.687, *df* = 9, CFI = 1, RMSEA = 0 (90% CI 0–0.050), SRMR = 0.027; Albanian: χ*^2^* = 8.207, *df* = 9, CFI = 0.997, RMSEA = 0.02 (90% CI 0–0.129), SRMR = 0.081]. In other words, the factor structures, loadings, and intercepts were equivalent for males and females within migrant groups (see Supplementary material S1).

Measurement invariance was also tested separately for negative attitudes of smoking across migrant groups at T1 and T2. Changes in χ*^2^* from configural to metric invariance were significant. In other words, the factor structures differed for Turkish- and Albanian-speaking persons. Whereas Turkish-speaking persons saw the biggest disadvantage of smoking in its aging effect of the skin, Albanian-speaking persons saw the biggest disadvantage of smoking in their damages to their health. Based on these results, further invariance testing was performed for both migrant groups separately. Measurement invariance for negative attitudes toward smoking was also not given for Turkish-speaking and Albanian-speaking women and men. Lastly, measurement invariance was tested for males and females separately for both groups, but no invariance could be identified over time (see Supplementary material S2). Due to the missing measurement invariance of negative attitudes toward smoking over time, no latent change score model was specified to examine associations between changes in negative attitudes and smoking behavior.

### Latent changes in positive attitudes and smoking behavior

**Figure 2:**
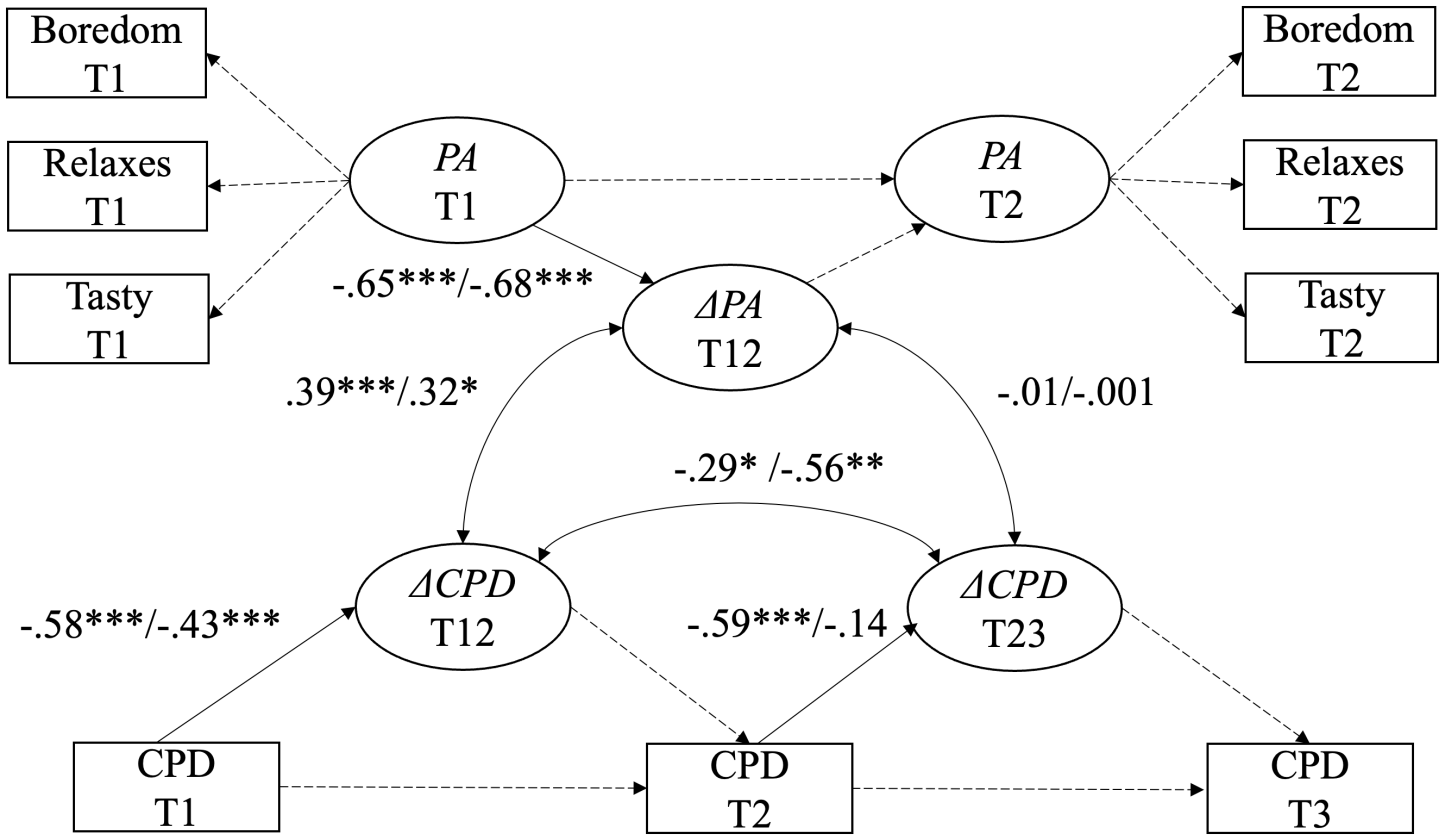
Latent change score model for changes in positive attitudes (PA) and its associations with changes in cigarettes smoked per day (CPD) between pre- (T1), post- (T2), and follow-up (T3) assessment. Auto-correlations between indicators, restrictions for variances and intercepts are not represented. Standardized estimates are displayed. Δ symbolizes latent change scores. All effects for Turkish-speaking people are displayed first, effects for Albanian-speaking people second. ^†^*p* = 0.10, ^*^p < 0.05, ^**^p < 0.01, ^***^p < 0.001.

For each of the two migrant groups (Turkish- and Albanian-speaking people), we specified a latent change score model examining positive attitudes toward smoking and their association with changes in smoking behavior over time, imposing strong measurement invariance (scalar) across time points. The models for the Turkish- and the Albanian-speaking group indicated an acceptable fit (Turkish: χ*^2^* = 36.06, *df* = 21, *p* = 0.022; CFI = 0.97; RMSEA = 0.06 (90% CI 0.02–0.09), *p* = 0.28; SRMR = 0.07. Albanian: χ *^2^* = 25.07, *df* = 21, *p* = 0.244; CFI = 0.96; RMSEA = 0.05 (90% CI 0.00–0.12), *p* = 0.44; SRMR = 0.09; all robust measures; complete cases: Turkish: *N* = 198; Albanian: *N* = 70). Figure 2 illustrates the results of both change score models.

Positive attitudes toward smoking decreased significantly from baseline to 3-months follow-up (Turkish: *β* = −0.65, *p* < 0.001; Albanian: *β* = −0.68, *p* < 0.001). Similarly, Turkish- and Albanian-speaking persons reduced the number of cigarettes smoked per day from baseline to 3-months follow-up (Turkish: *β* = −0.58, *p* < 0.001; Albanian: *β* = −0.43, *p* < 0.001). From 3-months to 1 year follow-up, Turkish-speaking persons further reduced the number of cigarettes (Turkish: *β* = −0.59, *p* < 0.001), while Albanian speaking persons showed no significant decrease (Albanian: *β* = −0.14, *p* = 0.57).

Latent correlations between change scores indicated that Turkish-as well as Albanian speaking persons who showed a decrease in positive attitudes also showed a decrease in cigarettes smoked per day from baseline to 3-months follow-up (Turkish: *r* = 0.39, *p* < 0.001; Albanian: *r* = 0.32, *p* = 0.03). However, the change in positive attitude between baseline and 3-months follow-up was not associated to a change in smoking behavior in the long term (Turkish: *r* = −0.01, *p* = 0.88; Albanian: *r* = −0.001, *p* = 0.99). Lastly, we found a negative association between the reduction of cigarettes smoked per day from baseline to 3-months follow-up and the reduction of cigarettes smoked from 3-month assessment to follow-up (Turkish: *r* = −0.29, *p* = 0.04; Albanian: *r* = −0.56, *p* = 0.006) indicating that the reduction in the short-term was not associated with the further reduction in the long-term.

### Sensitivity analysis

Due to the expiration of funds, data collection had to be suspended between T2 and T3 for a part of the sample and resumed to a later time point. As a consequence, the 1-year follow-up measure (T3) was assessed in mean 7.7 (*SD* = 1.1, range = 6–9) months later for 16 out of 70 Albanian-speaking and in mean 7.1 (*SD* = 1.5 range = 5–10) months later for 43 out of 198 Turkish-speaking migrants in the final sample. Hence, these participants had to recall how many cigarettes they smoked per day at T3. To preclude the possibility that results may be influenced by memory effects, we additionally collected the number of cigarettes smoked at the time of data collection (which was a longer time period than originally planned) for these participants. As a robustness check, we used this actual number of cigarettes at T3 in the latent change score model, which did not change the model fit or the pattern of results to a noticeable degree.

At the same time, a part of the follow-up assessment took place during the COVID-19 pandemic, which could have influenced smoking habits. To check the robustness of our results against an influence of the pandemic, we used a dichotomous indicator of whether the number of cigarettes smoked at T3 was assessed during the COVID-19 pandemic as a moderator on the estimates associated with ΔCPD T23 (correlations between ΔPA T12 and ΔCPD T23), between ΔCPD T12 and ΔCPD T23, and the regression of ΔCPD T23 on CPD T2. The fit for this adapted model decreased (Turkish: χ*^2^* = 121.54, *df* = 72, *p* < 0.001; CFI = 0.90; RMSEA = 0.08; SRMR = 0.13; Albanian: χ*^2^* = 82.34, *df* = 72, *p* = 0.190; CFI = 0.91; RMSEA = 0.07; SRMR = 0.16). The pattern of results remained unchanged, but two effects in each subgroup was no longer significant: the correlation between ΔCPD T12 and ΔCPD T23 was not significant anymore for the Turkish-speaking sample, and the regression of ΔCPD T23 on CPD T2 was significant for participants assessed during the Covid-19 pandemic in the Albanian-speaking sample.

## Discussion

### Principle findings

Using a proactively recruited sample of Turkish- and Albanian-speaking migrants living in Switzerland, the current study examined (1) the effects of an adapted smoking cessation course on attitudes toward smoking and smoking behavior; and (2) the associations between changes in attitudes toward smoking and changes in smoking behavior right after and 1-year after course completion. The main findings are: (1) Participation in an adapted smoking cessation course led to a decrease of positive attitudes toward smoking and a decrease of cigarettes smoked per day in the short-term for both migrant groups, and in the long-term for the Turkish-speaking migrants; and (2) greater decreases in positive attitudes were associated with greater reductions of smoking in the short-term for both migrant groups, but not in the long-term.

The results of this study are in line with Nierkens et al. (2005) findings on the predictive role of advantages of smoking for explaining smoking cessation in former migrant smokers. However, they do go a step further than their results and those findings from other studies on non-migrants (Kramer Lafferty et al., 1999; Etter et al., 2000), by establishing this relationship from prospectively collected, longitudinal data. This study is also the first to show how changes in positive attitudes, most likely stimulated by the attendance of an adapted smoking cessation course, were associated with intended behavior change in the short-term.

In this study, changes in positive attitudes were not associated with long-term smoking reductions in neither migrant group. These results correspond with those from studies based on the Health Action Process Approach (HAPA), which found that attitudes is one of the main components for forming an behavior change intention, while other socio-cognitive characteristics, such as self-efficacy, action control and planning, are decisive for behavior change maintenance (Schwarzer, 1992; Scholz et al., 2009). However, further studies will have to examine, if the long-term reduction in smoking, as shown by the Turkish-speaking participants who took part in the adapted smoking cessation courses, were triggered by changes in socio-cognitive characteristics associated with behavior maintenance. Future studies should also assess if the lack of smoking reduction in the long-term, as illustrated by the Albanian-speaking participants, was in turn associated with deficient behavior maintenance strategies or rather with characteristics of the subgroup and their living situation. If the lack of long-term reductions in Albanian-speaking migrants were related to the treatment not being able to change cognitions relevant to maintenance in this subgroup, group-specific modifications of the last three sessions dedicated to relapse prevention would be the consequence. However, as illustrated by the sensitivity analyses in this paper, it seems more plausible that the lack of long-term reductions in this subgroup were related to Albanian-speaking participants being more likely to experience a relapse due to the COVID-19 pandemic situation.

Additionally, this study further highlights the need for invariance testing across different populations and groups. As the results of this study show, migrant populations are diverse with respect to their attitudes toward smoking, and this has to be considered in practice and research. This means that other cessation courses will be especially effective if they tailor their content to the most salient advantage of smoking for the specific migrant group. For Turkish-speaking people this could mean to find more appropriate ways to relax, whereas for Albanian-speaking people this would entail to look for alternatives against boredom. Specifically, the failure to establish measurement invariance for negative attitudes toward smoking across and within groups in our sample highlights the relevance of this methodology. Studying changes in negative attitudes toward smoking in this sample would have led to meaningless results and perhaps to erroneous conclusions about the treatment effectiveness. Future studies should however go a step further, and explain the absence of measurement invariance in negative attitudes toward smoking in migrant groups, for example following the work of Davidov et al. using multilevel equation modeling (Davidov et al., 2018). In their study, the authors were able to explain missing scalar equivalence in a scale measuring attitudes toward granting citizenship rights to migrants across different countries in part by the percentage of foreigners living in the countries itself. In our study, the items measuring positive attitudes toward smoking (“Smoking helps against boredom/calms and relaxes/tastes good”) relate to the smoking person themselves. In contrast, the items measuring negative attitudes relate to people in the surrounding or the impression created on those people (“Smoking leaves an unpleasant smell/makes the skin age faster/damages the health of other people”). In this light, measurement invariance for negative attitudes may be due to differences in which aspects are important when trying to leave a positive impression on others (see Schlenker and Weigold, 1992) that may be different between groups and gender.

### Strengths and limitations

Strengths of this study include our testing for measurement invariance in attitudes toward smoking and the several sensitivity analyses, which suggest that the results are robust. Notable limitations are the relatively high proportion of missing information at the different time points and the higher probability of participants with lower ratings on positive attitudes to be assessed at 3-month follow-up. For the 3-month and 1-year follow-up assessments, we had to rely on self-reports, which is the most notable limitation of this study. To minimize under-reporting of tobacco use, trained interviewers who were unknown to participants conducted the telephone assessments. Lastly, in this article we focused on the relationship between attitude change and smoking behavior change. At the same time, inter-individual differences with respect to absolute level of smoking (the number of cigarettes per day) could also impact smoking cessation behavior (Paz Castro et al., 2021). Future research could investigate whether the relationship between attitude change and smoking behavior change differs between smokers at different levels.

### Conclusion

Adapted smoking cessation courses fostered changes in positive attitudes toward smoking that were associated with intended behavior change in the short-term. Turkish-speaking—but not Albanian-speaking participants—showed further long-term smoking reductions, but changes in positive attitudes were not associated with long-term behavior changes. Future studies need to examine the role of socio-cognitive characteristics related to behavior change maintenance to inversely allow conclusions about the long-term effectiveness of the adapted smoking cessation courses and about the need for further adaptations.

## Data availability statement

The data that support the findings of this study are openly available in SwissUbase at: https://www.swissubase.ch/en/catalogue/studies/14036/16989/datasets.

## Ethics statement

Ethical review and approval was not required for the study on human participants in accordance with the local legislation and institutional requirements. Written informed consent for participation was not required for this study in accordance with the national legislation and the institutional requirements.

## Author contributions

MS and CSG contributed to the design and implementation of the research. RPC and MH to the analysis of the results and writing of the manuscript. All authors contributed to the article and approved the submitted version.

## Funding

This work was supported by the Tobacco Control Fund at Federal Office of Public Health Switzerland.

## Conflict of interest

The authors declare that the research was conducted in the absence of any commercial or financial relationships that could be construed as a potential conflict of interest.

## Publisher’s note

All claims expressed in this article are solely those of the authors and do not necessarily represent those of their affiliated organizations, or those of the publisher, the editors and the reviewers. Any product that may be evaluated in this article, or claim that may be made by its manufacturer, is not guaranteed or endorsed by the publisher.
