# Supplementary material for: Changes in attitudes towards smoking during smoking cessation courses for Turkish- and Albanian-speaking migrants in Switzerland and its association with smoking behavior: A latent change score approach
Source: Front Psychol. 2022 Dec 22;13:1032091. doi: 10.3389/fpsyg.2022.1032091 (PMC9813416; doi:10.3389/fpsyg.2022.1032091)
Supplement: Supplementary file 1 [file Table_1.DOCX]

| Supplementary material 1. Fit indices for measurement invariance of positive attitudes towards smoking across migrant groups, gender within migrant groups, and over time | | | | | | | | | | |
| --- | --- | --- | --- | --- | --- | --- | --- | --- | --- | --- |
|  | χ*^2^* | *df* | Δχ*^2^* | Δ*df* | CFI | ΔCFI | RMSEA | RSMEA 90% CI | SRMR | Invariance? |
| Across migrant groups at T1 (n=358) | | | | | | | | |  |  |
| configural | 0 | 0 |  |  | 1 |  | 0 | 0-0 | 0 |  |
| metric | 19.106 | 3 | 19.106 | 3 | 0.944 | 0.056 | 0.212 | 0.144-0.289 | 0.076 | NO |
| scalar |  |  |  |  |  |  |  |  |  |  |
| Across migrant groups at T2 (n=336) | | | | |  |  |  |  |  |  |
| configural | 0 | 0 |  |  | 1 |  | 0 | 0-0 | 0 |  |
| metric | 1.992 | 3 | 1.992 | 3 | 1 | 0 | 0 | 0-0.124 | 0 | YES |
| scalar | 2.906 | 1 | 0.914 | 2 | 1 | 0 | 0 | 0- 0.188 | 0.034 | YES |
| Across gender within Turkish-speaking group at T1 (n=230) | | | | | | |  |  |  |  |
| configural | 0 | 0 |  |  | 1 |  | 0 | 0-0 | 0 |  |
| metric | 0.462 | 3 | 0.462 | 3 | 1 | 0 | 0 | 0-0.033 | 0.015 | YES |
| scalar | 1.082 | 1 | 0.620 | 2 | 1 | 0 | 0.027 | 0-0.251 | 0.04 | YES |
| Across gender within Turkish-speaking group at T2 (n=231) | | | | | | |  |  |  |  |
| configural | 0 | 0 |  |  | 1 |  | 0 | 0-0 | 0 |  |
| metric | 5.991 | 3 | 5.991 | 3 | 0.979 | 0.021 | 0.127 | 0.029-0.231 | 0.063 | YES |
| scalar | 8.849 | 1 | 2.858 | 2 | 0.988 | -0.009 | 0.17 | 0.033-0.348 | 0.075 | YES |
| Across gender within Albanian-speaking group at T1 (n=126) | | | | | | |  |  |  |  |
| configural | 0 | 0 |  |  | 1 |  | 0 | 0-0 | 0 |  |
| metric | 8.736 | 4 | 8.736 | 4 | 0.963 | 0.037 | 0.165 | 0.047-0.288 | 0.08 | YES |
| scalar | 8.736 | 1 | 0.0004 | 3 | 0.981 | -0.018 | 0.233 | 0.048-0.474 | 0.08 | YES |
| Across gender within Albanian-speaking group at T2 (n=102) | | | | | | | | |  |  |
| configural | 0 | 0 |  |  | 1 |  | 0 | 0-0 | 0 |  |
| metric | 7.903 | 4 | 7.903 | 4 | 0.925 | 0.075 | 0.145 | 0-0.287 | 0.124 | YES |
| scalar | 7.903 | 1 | 7.903 | 3 | 0.962 | -0.037 | 0.205 | 0-0.482 | 0.124 | YES |
| Over time for Turkish-speaking group (n=231) | | | | | |  |  |  |  |  |
| configural | 0.749 | 5 |  |  | 1 |  | 0 | 0-0.034 | 0.016 |  |
| metric | 1.032 | 5 | 0.283 | 0 | 1 | 0 | 0 | 0-0 | 0.016 | YES |
| scalar | 3.687 | 9 | 2.655 | 4 | 1 | 0 | 0 | 0-0.050 | 0.027 | YES |
| Analyzed sample (n=202) | |  |  |  |  |  |  |  |  |  |
| scalar | 3.956 | 1 |  |  | 0.989 |  | 0.173 | 0.017-0.366 | 0.069 | YES |
| Over time for Albanian-speaking group (n=102) | | | | | |  |  |  |  |  |
| configural | 2.886 | 5 |  |  | 1 |  | 0 | 0-0.133 | 0.050 |  |
| metric | 5.337 | 5 | 2.451 | 0 | 1 | 0 | 0 | 0-0.149 | 0.065 | YES |
| scalar | 8.207 | 9 | 2.87 | 4 | 0.997 | 0.003 | 0.020 | 0-0.129 | 0.081 | YES |
| Analyzed sample (n=70) | |  |  |  |  |  |  |  |  |  |
| scalar | 3.541 | 1 |  |  | 0.957 |  | 0.273 | 0-0.605 | 0.071 | YES |

*Note*. χ*^2^* = Chi-square; df= degree of freedom; Δχ*^2^* = Chi-square difference; Δ*df* = change in degree of freedom; CFI = Comparative fit ind ex; ΔCFI = CFI change; RMSEA = root mean square error of approximation; 90% CI = 90% Confidence interval of RMSEA; SRMR = Standardized root mean square residuals
