# Supplementary material for: Changes in attitudes towards smoking during smoking cessation courses for Turkish- and Albanian-speaking migrants in Switzerland and its association with smoking behavior: A latent change score approach
Source: Front Psychol. 2022 Dec 22;13:1032091. doi: 10.3389/fpsyg.2022.1032091 (PMC9813416; doi:10.3389/fpsyg.2022.1032091)
Supplement: Supplementary file 2 [file Table_2.DOCX]

| Supplementary material S2. Fit indices for measurement invariance of negative attitudes towards smoking across migrant groups, gender within migrant groups, and over time | | | | | | | | | | |
| --- | --- | --- | --- | --- | --- | --- | --- | --- | --- | --- |
|  | χ*^2^* | *df* | Δχ*^2^* | Δ*df* | CFI | ΔCFI | RMSEA | RSMEA 90% CI | SRMR | Invariance? |
| Across migrant groups at T1 (n=383) | | | | | | | | |  |  |
| configural | 0 | 0 |  |  | 1 |  | 0 | 0-0 | 0 |  |
| metric | 45.668 | 3 | 45.668 | 3 | 0.719 | 0.281 | 0.328 | 0.261-0.400 | 0.219 | NO |
| scalar |  |  |  |  |  |  |  |  |  |  |
| Across migrant groups at T2 (n=334) | | | | |  |  |  |  |  |  |
| configural | 0 | 0 |  |  | 1 |  | 0 | 0-0 | 0 |  |
| metric | 21.098 | 3 | 21.098 | 3 | 0.715 | 0.285 | 0.265 | 0.193-0.343 | 0.138 | NO |
| scalar |  |  |  |  |  |  |  |  |  |  |
| Across gender within Turkish-speaking group at T1 (n=244) | | | | | | |  |  |  |  |
| configural | 0 | 0 |  |  | 1 |  | 0 | 0-0 | 0 |  |
| metric | 13.405 | 4 | 13.405 | 3 | 0.861 | 0.139 | 0.151 | 0.075-0.236 | 0.422 | NO |
| scalar |  |  |  |  |  |  |  |  |  |  |
| Across gender within Turkish-speaking group at T2 (n=231) | | | | | | |  |  |  |  |
| configural | 0 | 0 |  |  | 1 |  | 0 | 0-0 | 0 |  |
| metric | 18.737 | 3 | 18.737 | 3 | 0.875 | 0.125 | 0.192 | 0.116-0.277 | 0.159 | NO |
| scalar |  |  |  |  |  |  |  |  |  |  |
| Across gender within Albanian-speaking group at T1 (n=137) | | | | | | |  |  |  |  |
| configural | 0 | 0 |  |  | 1 |  | 0 | 0-0 | 0 |  |
| metric | 21.75 | 4 | 21.75 | 4 | 0.944 | 0.056 | 0.277 | 0.179-0.386 | 0.141 | NO |
| scalar |  |  |  |  |  |  |  |  |  |  |
| Across gender within Albanian-speaking group at T2 (n=100) | | | | | | | | |  |  |
| configural | 0 | 0 |  |  | 1 |  | 0 | 0-0 | 0 |  |
| metric |  |  |  |  |  |  |  |  |  | NO* |
| scalar |  |  |  |  |  |  |  |  |  |  |
| Turkish-speaking women over time (n=78) | | | | | |  |  |  |  |  |
| configural |  |  |  |  |  |  |  |  |  | NO* |
| metric |  |  |  |  |  |  |  |  |  |  |
| scalar |  |  |  |  |  |  |  |  |  |  |
| Turkish-speaking men over time (n=59) | | | | | |  |  |  |  |  |
| configural |  |  |  |  |  |  |  |  |  | NO* |
| metric |  |  |  |  |  |  |  |  |  |  |
| scalar |  |  |  |  |  |  |  |  |  |  |
| Albanian-speaking women over time (n=55) | | | | | | | | | | |
| configural |  |  |  |  |  |  |  |  |  | NO* |
| metric |  |  |  |  |  |  |  |  |  |  |
| scalar |  |  |  |  |  |  |  |  |  |  |
| Albanian-speaking men over time (n=45) | | | | | |  |  |  |  |  |
| configural |  |  |  |  |  |  |  |  |  | NO* |
| metric |  |  |  |  |  |  |  |  |  |  |
| scalar |  |  |  |  |  |  |  |  |  |  |

*Note*. χ*^2^* = Chi-square; df= degree of freedom; Δχ*^2^* = Chi-square difference; Δ*df* = change in degree of freedom; CFI = Comparative fit ind ex; ΔCFI = CFI change; RMSEA = root mean square error of approximation; 90% CI = 90% Confidence interval of RMSEA; SRMR = Standardized root mean square residuals; *the model could not converge.
